# Association of early parent–child separation with depression, social and academic performance in adolescence and early adulthood: a prospective cohort study

**DOI:** 10.1186/s13034-024-00769-1

**Published:** 2024-06-26

**Authors:** Honghua Li, Kai Liu, Junsong Fei, Tongshuang Yuan, Songli Mei

**Affiliations:** 1 Department of Developmental and Behavioral Pediatrics, Children’s Medical Center, The First Hospital of Jilin University, No. 1 Xinmin Street, Changchun, 130021 Jilin Province China; 2 Department of Social Medicine and Health Management, School of Public Health, Jilin University, No.1163 XinMin street, Changchun, 130021 Jilin Province China

**Keywords:** China Family Panel Studies, Infancy and early childhood, Parent–child separation, Depression, Social relationship, Academic performance

## Abstract

**Objective:**

The present study aimed to investigate the long-term effects of parent–child separation during infancy and early childhood on depression, social relationships including parent–child and peer relationships, and academic performance during adolescence and early adulthood.

**Methods:**

Data from the China Family Panel Studies (CFPS) were analyzed, which included a sample of 3829 children aged 4–15 years from 25 provinces over a period of 8 years. The study examined the association between early parent–child separation and outcomes related to depression, social and academic performance, comparing outcomes between individuals with and without early separation experiences. A series of subgroup analyses were conducted to further explore these associations.

**Results:**

Parent–child separation lasting 3 months or longer was found to be associated with moderate to severe levels of depression and impaired social relationships during adolescence and early adulthood, particularly among males, adolescents, urban dwellers, and those with less educated mothers. Children who experienced parent–child separation for 3 months or longer showed a positive correlation between separation duration and depression. Short-term separations under 3 months did not show this association. The duration of separation also had a negative correlation with parent–child and peer relationships, as well as academic performance.

**Conclusion:**

Early parent–child separation has significant adverse effects on the mental health, social and academic performance of adolescents and early adulthood, especially among males, adolescents, urban residents, and those with lower maternal education. The severity of depression was found to be related to the duration of separation, highlighting the importance of minimizing separation to less than 3 months for children under the age of 3. These findings underscore the critical role of early parental care and the need for targeted interventions for high-risk populations.

**Supplementary Information:**

The online version contains supplementary material available at 10.1186/s13034-024-00769-1.

## Introduction

A stable family structure is crucial for the healthy development of children [1]. Economic globalization has led to an increase in migration worldwide. In China, industrialization and urbanization have caused a significant movement of population. Many people have migrated from rural to urban areas or between different cities in search of better employment opportunities [2]. However, due to institutional barriers, particularly the household registration (hukou) system, urban residents with urban household registration enjoy more social welfare benefits compared to rural residents with rural household registration, such as healthcare and education for their children. Meanwhile, migrants are required to meet certain criteria, such as stable employment or property ownership, to obtain urban household registration, which restricts their access to urban social welfare. Consequently, parents are often compelled to leave their children in their hometowns for extended periods and entrust their care to extended family members, typically grandparents [3]. Based on the data collected from the seventh national census conducted in 2020, approximately 41.77 million rural children and 25.16 million urban children in China have experienced parent–child separation, accounting for about 22.5% of the child population in China [4]. Although labor migration alleviates family economic conditions, the adverse impact of parent–child separation induced by such migration on children's long-term health outcomes should not be underestimated, especially when the separation occurs in early stages of life.

The period from 0 to 3 years is recognized as a critical period for brain and emotional developments in children [5]. Establishing a secure attachment relationship between children and parents during this period is crucial for children's emotion, social and academic development [6, 7]. Moreover, developmental theories emphasize the significant impact of early experiences on individual development [8]. Conversely, parent–child separation during this period disrupts parenting practices and interactions, potentially leading to insecurity and ambiguous loss for children, which may contribute to adverse outcomes in mental health, social and academic achievement [1, 8–10]. Additionally, grandparents' parenting style and physical condition may limit their involvement and patience, affecting children's emotional regulation and the development of social skills [11, 12]. Over time, these factors may lead to a series of challenges in the health and well-being of children in the later life. Emotional well-being, social relationships including parent–child and peer relationships, and academic performance are key aspects of social adaptation during adolescence and early adulthood. Research implies that early parent–child relationships foster the development of social skills that are essential for the establishment of meaningful peer relationships [13]. Conversely, an unstable caregiving environment resulting from parent–child separation inhibit the acquisition of social skills in children, subsequently affecting parent–child and peer relationships [14]. Additionally, separation from parents may also impact adolescents' academic performance [14, 15].

Existing research indicates that parent–child separation, as an early adverse life event, is associated with poorer mental and physical health outcomes during adolescence, as well as limitations in social skills and emotional development [14, 16, 17]. Despite this, the life course perspective emphasizes the timing of life events influencing later life outcomes [18]. Therefore, it is important to consider that different timings of parent–child separation may lead to variations in later life outcomes. The association between parent–child separation during the ages of 4 to 15 and depression in adolescents and young adults has been examined by Zhao et al. [19, 20]. They observed that the association was more pronounced among older individuals and those residing in rural areas. In contrast, Yang et al. [21] did not find age-related differences in the relationship between parental absence before the age of 3 and later-life depression. However, their study discovered that children living in urban areas who experienced parental absence before the age of 3 had a higher risk of developing depression compared to children in rural areas, with girls being more vulnerable than boys. These inconsistencies may be attributed to discrepancies in the timing of parent–child separation across different study populations. Lindström et al.'s research supports the notion that earlier separation from parents, particularly between 0 and 4 years old, may have a greater impact on psychological well-being in adulthood [22].

Fathers and mothers play different roles in individual development [23], and the traditional family model in China influences division of labor, with mothers primarily responsible for direct caregiving while fathers typically provide material support [24]. Research indicates that individuals with lower caregiver education levels are at higher risk of experiencing emotional and behavioral problems following parent–child separation [25]. Therefore, we hypothesize that the impact of parent–child separation on children's emotional and social development may vary depending on the mother's level of education. Additionally, parent–child separation due to marital conflicts has been linked to various academic difficulties, such as poor academic performance, truancy, and grade retention [26, 27]. However, Hou [28] found that parent–child separation resulting from rural-to-urban migration was associated with improved academic outcomes. These contradictory results may be attributed to differences in ethnicity, cultural background, and the underlying reasons for parent–child separation, with labor migration being the primary cause of separation in China. Given the mixed findings from previous research, it is necessary to clarify the effects of early parent–child separation on later depression, social and academic performance, considering different factors such as gender, age group, place of residence, and maternal education level.

In summary, there have been limited studies investigating the long-term effects of early parent–child separation on children's mental health, social and academic performance in China, particularly when the separation occurred at or before the age of 3. According to the theory of cumulative disadvantage, the duration of separation may have varying impacts on adolescent health outcomes [29]. Furthermore, existing studies in this field have yielded inconsistent findings, highlighting the need for cohort studies to explore the associations and potential causal relationships between early parent–child separation, the duration of separation, and later outcomes such as depression, social and academic performance in children. This will contribute to a better understanding of the long-term effects of parent–child separation on later outcomes.

To ensure the reliability of the research, we selected several covariates at the individual, parental, and family levels, including individual gender, age, place of residence, educational stage, school location, parental education level, family size, and economic status. Previous research has suggested that these variables may have potential influences on the health outcomes [10, 20, 21]. Therefore, this study intends to control for these variables to enhance the internal validity of the research.

The China Family Panel Studies (CFPS) have conducted comprehensive assessments on depression, social and academic performance in a national representative sample of the Chinese population and gathered data on demographic characteristics and the living environment of each household [30]. Building upon prior research, the present study proposes three hypotheses: (1) the experience of early parent–child separation is positively associated with depression and negatively associated with social and academic performance when children enter adolescence or early adulthood, compared to those without separation experience; (2) the effects of early parent–child separation on depression, social and academic performance later in life may differ based on factors such as gender, age groups, residence, and maternal education levels; and (3) the duration of early parent–child separation is positively correlated with the severity of depression and negatively correlated with social and academic performance later in life. By investigating above hypotheses, the study aims to provide further insights into the long-term consequences of early parent–child separation on mental health and development of children in the Chinese context.

## Methods

### Participants and procedure

**Fig. 1:**
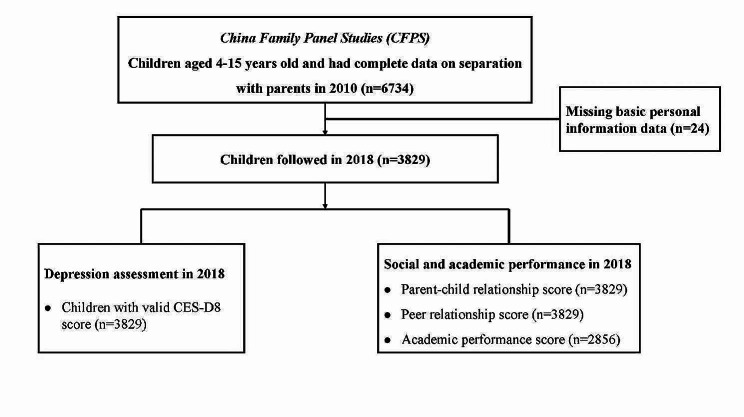
A flowchart of participant selection

The data used in this study was obtained from the CFPS, a publicly available database conducted by the Institute of Social Science Survey at Peking University [30]. The CFPS is a nationally representative longitudinal survey that covers 25 provinces in China, representing approximately 95% of the national population. Using a multi-stage probability-proportional-to-size sampling method, the administrative divisions of China were first divided into geographical units, followed by the random selection of streets or villages within each unit. Subsequently, a certain number of households were randomly chosen within each street or village, and all members of these households were surveyed. Weight allocation was also conducted based on geographical locations and population characteristics. The CFPS randomly selected 57,152 participants, including 8990 children who participated in the survey. The baseline survey of the CFPS began in April 2010, with follow-ups conducted every 2 years until 2020. To investigate the impact of early parent–child separation on depression, social and academic performance in adolescence and early adulthood, this study utilized data from the 2010 survey as the baseline. This dataset included 6734 children aged 4 to 15 years old, with detailed information on parent–child separation available. Among them, 3829 (56.9%) children were successfully followed up in 2018 (Fig. 1). The Peking University Biomedical Ethics Review Committee approved the survey (Approval number: IRB00001052-14010). All participants in the CFPS survey were required to sign an informed consent form before the interview (https://www.isss.pku.edu.cn/cfps/xgxw/cfpsdt/1357060.htm, accessed on Nov 19, 2023).

### Measures

#### Early parent–child separation

In the present study, parent–child separation was defined as the duration of continuous separation from both parents occurring either at or before the child reached the age of 3. This information was reported by parents in the baseline survey of 2010, where interviewers asked them: "How many consecutive weeks were both parents separated from their children at or before the age of 3?" Based on the responses to this question, we measured the duration of parent–child separation during infancy and early childhood and divided the participants into two categories: unseparated (81.6%) and parent–child separated (18.4%). Based on the distribution of separation duration and the non-linear relationship observed between separation duration and depression scores, a change in the curve's direction was observed at the turning point of 3 months (Figure S1). Consequently, the latter category was further divided into two subcategories: separation for less than 3 months (Separation < 3 m) and separation for 3 months or longer (Separation ≥ 3 m).

#### Depression

In the 2018 CFPS survey, a simplified version of the Center for Epidemiologic Studies Depression Scale (CES-D) consisting of eight items was utilized to evaluate depression status [31–33]. The CES-D8 consists of six negative and two positive statements, namely: (1) feeling down in spirits; (2) finding it challenging to do everything; (3) experiencing poor sleep; (4) feeling lonely; (5) feeling sad; (6) feeling hopeless; (7) feeling fine; and (8) leading a happy life. Participants were asked to rate the frequency of these feelings or actions over the past week using a four-point Likert scale: "scarcely any (< 1 day)", "sometimes (1–2 days)", "frequently or often (3–4 days)", and "most of the time (5–7 days)". The two items representing positive feelings were reverse-scored. The score for each item was summed to obtain a total score, which should fall within the range of 0 to 24. A higher total score indicates a more severe level of depressive symptoms for the participant. Briggs et al. [34] suggests that a total score of 9 or higher on the CES-D8 indicates clinically significant depressive symptoms in individuals. The CES-D has been extensively employed and validated in various studies and populations [32, 33, 35, 36]. In this study, the Cronbach's alpha coefficient for the CES-D8 was 0.737.

#### Social and academic performance

Social and academic performance are important aspects of adolescent social adaptation [37, 38]. Parent–child and peer relationships serve as crucial social bonds for adolescents [37]. The level of trust adolescents have in their parents is closely related to the quality of the parent–child relationship [39]. In the 2018 CFPS survey, adolescents were asked to rate their level of trust in their parents on a scale ranging from 0 (complete distrust) to 10 (complete trust). Higher scores indicate a more positive parent–child relationship. Additionally, the survey included the question "How good are your relationships with peers?" to assess the quality of peer relationship, with scores ranging from 0 to 10. Higher scores indicate better relationship with peers. The measurement of academic performance can be divided into objective academic performance and subjective self-evaluation [40]. In this study, we used adolescents' subjective self-evaluations to measure academic performance. Another survey question was "How would you rate your academic performance?" with response options ranging from 1 (very dissatisfied/very poor) to 5 (very satisfied/very good). Higher scores indicate better academic performance. Previous research has supported the reliability of using single-item measures to assess social and academic performance [40–42].

#### Covariates

Covariates were selected based on relevant research and systematic reviews [10, 20, 21], including characteristics of children, characteristics of parents, and household-level characteristics (Table 1). Characteristics of children include their gender, age, residence, ethnicity, gestation, birthweight, Body mass index (BMI), educational stage in 2018, school location, and academic stress. Characteristics of parents include their ages, educational levels, and marital status. Family-level characteristics investigate the family size and net household income per capita in 2018.

**Table 1:** Characteristics and comparison of the study population

Characteristics	Total (N = 3829)		No separation (N = 3125)		Separation < 3 m (N = 306)		Separation ≥ 3 m (N = 398)		*F(H)*/*χ2*
	N	M ± SD/*n *(%)	N	M ± SD/*n *(%)	N	M ± SD/*n *(%)	N	M ± SD/*n *(%)	
Gender	3829		3125		306		398		5.57
Male		2018 (52.7%)		1674 (53.6%)		154 (50.3%)		190 (47.7%)	
Female		1811 (47.3%)		1451(46.4%)		152 (49.7%)		208 (52.3%)	
Age in 2010, years	3829	9.2 ± 3.5	3125	9.3 ± 3.5	306	8.9 ± 3.6	398	8.2 ± 3.3	19.86***
Age in 2018, years	3829	17.2 ± 3.5	3125	17.3 ± 3.5	306	16.9 ± 3.6	398	16.2 ± 3.3	20.11***
Residence	3829		3125		306		398		1.42
Rural		1965 (51.3%)		1602 (51.3%)		150 (49%)		213 (53.5%)	
Urban/town		1864 (48.7%)		1523 (48.7%)		156 (51%)		185 (46.5%)	
Ethnicity	3829		3125		306		398		56.56***
Han		3384 (88.4%)		2797 (89.5%)		230 (75.2%)		357 (89.7%)	
other		445 (11.6%)		328 (10.5%)		76 (24.8%)		41 (10.3%)	
Gestation, months	3793	9.3 ± 0.6	3100	9.3 ± 0.6	305	9.3 ± 0.6	388	9.3 ± 0.7	1.71
Birthweight, kg	3115	3.2 ± 0.6	2548	3.2 ± 0.6	262	3.1 ± 0.5	305	3.1 ± 0.6	1.81
BMI at 2010 (kg / m^2^)	3560	17.8 ± 4.9	2902	17.8 ± 4.8	296	18.4 ± 5.8	362	17.4 ± 5.1	3.56*
Education stage in 2018	2856		2291		236		329		23.32***
Primary school		523 (18.3%)		400 (17.5%)		46 (19.5%)		77 (23.4%)	
Middle school		1042 (36.5%)		813 (35.5%)		98 (41.5%)		131 (39.8%)	
High school /technical school		748 (26.2%)		621 (27.1%)		46 (19.5%)		81 (24.6%)	
College or higher		543 (19.0%)		457 (19.9%)		46 (19.5%)		40 (12.2%)	
CES-D8 score in 2018, range 0–24	3829	4.6 ± 3.2	3125	4.5 ± 3.2	306	4.5 ± 3.3	398	4.9 ± 3.3	(5.65)
Social and academic performance in 2018									
Parent–child relationship, range 0–10	3829	9.4 ± 1.2	3125	9.5 ± 1.1	306	9.4 ± 1.1	398	9.2 ± 1.4	11.8***
Peer relationship, range 0–10	3829	7.1 ± 1.8	3125	7.1 ± 1.8	306	7.1 ± 1.7	398	6.9 ± 1.8	1.92
Academic performance, range 1–5	2856	3.3 ± 0.8	2291	3.3 ± 0.9	236	3.3 ± 0.8	329	3.2 ± 0.8	2.19
Academic stress in 2018, range 1–5	2856	3.0 ± 1.0	2291	3.0 ± 1.0	236	3.1 ± 1.1	329	3.0 ± 1.0	1.22
School location in 2018	2855		2290		236		329		19.92**
Provincial capital city		420 (14.7%)		353 (15.4%)		40 (16.9%)		27 (8.2%)	
Prefecture-level city		696(24.4%)		574 (25.1%)		52 (22.0%)		70 (21.3%)	
County seat		765 (26.8%)		601 (26.2%)		67 (28.4%)		97 (29.5%)	
Rural		974 (34.1%)		762 (33.3%)		77 (32.6%)		135 (41.0%)	
Parents' characteristics in 2010									
Paternal age	3810	37.4 ± 5.7	3111	37.8 ± 5.7	305	36.7 ± 5.9	394	34.9 ± 5.0	46.1***
Maternal age	3795	35.5 ± 5.5	3104	35.9 ± 5.5	304	34.7 ± 5.5	387	33.0 ± 5.2	52.3***
Paternal educational level	3762		3072		304		386		6.71
No formal education		578 (15.4%)		468 (15.2%)		58 (19.1%)		52 (13.5%)	
Primary school		973 (25.9%)		806 (26.2%)		69 (22.7%)		98 (25.4%)	
Middle school		1566 (41.6%)		1275 (41.5%)		120 (39.5%)		171 (44.3%)	
High school/Technical school		463 (12.3%)		374 (12.2%)		41 (13.5%)		48 (12.4%)	
College or higher		182 (4.8%)		149 (4.9%)		16 (5.3%)		17 (4.4%)	
Maternal educational level	3753		3078		299		376		22.87**
No formal education		978 (26.1%)		805 (26.2%)		95 (31.8%)		78 (20.7%)	
Primary school		1034 (27.6%)		861 (28.0%)		67 (22.4%)		106 (28.2%)	
Middle school		1299 (34.6%)		1040 (33.8%)		101 (33.8%)		158 (42.0%)	
High school/Technical school		294 (7.8%)		253 (8.2%)		20 (6.7%)		21 (5.6%)	
College or higher		148 (3.9%)		119 (3.9%)		16 (5.4%)		13 (3.5%)	
Parental marital status	3779		3092		302		385		7.15*
In marriage		3687 (97.6%)		3023 (97.8%)		296 (98.0%)		368 (95.6%)	
Divorced/Single/Widowed		92 (2.4%)		69 (2.2%)		6 (2.0%)		17 (4.4%)	
Family characteristics									
Family size in 2018	3829	4.4 ± 1.8	3125	4.4 ± 1.8	306	4.3 ± 1.6	398	4.7 ± 1.9	6.84**
Net household income per capita in 2018 (CNY)^a^	3172	12,330 (5,738, 22,987)	2576	12,675 (6000, 23,333)	264	10,711 (5487, 21,418)	332	10,293 (4215 19,654)	(13.11)**

CES-D8, Center for epidemiologic studies depression scale consisting of eight items; BMI, Body mass index; CNY, Chinese Yuan Renminbi; M, mean; SD, standard deviation

^a^Variable was described by percentile P50 (P25, P75). *P < 0.05; **P < 0.01; ***P < 0.001

### Statistical analysis

Data analysis was conducted using SPSS V24.0 (IBM, Armonk, NY, USA) and Stata software, version 14 (StataCorp LLC). Continuous variables were presented as mean and standard deviation (SD), while categorical variables were presented as frequencies and percentages. Univariate differences in individual, parental, and family characteristics among different groups were analyzed using one-way ANOVA, Kruskal–Wallis test, and Chi-square test, depending on the data distribution and type.

Quantile regression analysis was employed to examine the association between different categories of parent–child separation and children's levels of depression in later life. The analysis was performed at quantiles 0.25, 0.50, and 0.75. Regression coefficients and 95% confidence intervals (CI) were calculated, controlling for variables that showed significant differences in the univariate analysis. Moreover, multivariate linear regression analysis was conducted to explore the association between different categories of parent–child separation, social and academic performance later in life. Both unadjusted and fully adjusted models were tested, with control for individual, parental, and family-level characteristics in the latter. Subgroup analyses were then conducted to investigate the association between parent–child separation and children's depression, social and academic performance later in life across different gender, age groups, residence, and maternal educational level. Regression coefficients and 95% CI were calculated for each subgroup.

A restricted cubic spline (RCS) model was utilized to investigate the "dose–response" relationship between the duration of separation and depression, as well as social and academic performance. The RCS curve for the duration of separation and depression score was plotted with the reference value set at P10, using four percentiles (P5, P35, P65, P90) as knots (the number of knots was selected based on the lowest Akaike information criterion value). Similarly, the RCS curve for the duration of parent–child separation and social and academic performance was plotted using the same RCS model, with the reference value set at P10 and three percentiles (P10, P50, P90) used as knots. If the RCS curve exhibited a U-shape, the inflection point (where the curve changed direction) was identified as a cut-off value. The data was then divided into two segments based on this cut-off value, and segmented linear regression was performed. If the curve approximated a straight line, multivariate linear regression was conducted. RCS curve plotting was performed using R software version 4.2.2, along with the rms package and MSTATA software. A (two-sided) *P*-value of less than 0.05 was considered statistically significant.

## Results

### Characteristics of the study sample

Table 1 presents the sociodemographic characteristics and aggregated statistics of the children, parents, and families included in this study. A total of 3829 children aged 4 to 15 years participated in the 8-year follow-up assessment. Among them, 704 (18.4%) children experienced separation from their parents at 3 years old or younger, with an average duration of separation of 7.7 ± 8.7 months. We conducted a comparative analysis between the group of children with no parent–child separation and the groups with separation < 3 m and separation ≥ 3 m. In the separation ≥ 3 m group, the average age at baseline for both the children and their parents were significantly younger compared to the group without separation experience. Moreover, this group exhibited a larger family size coupled with a lower per capita net household income (Table 1).

In the 2018 follow-up assessment, scores on parent–child relationship were significantly lower in the group of children who experienced a separation ≥ 3 m compared to children who did not experience separation. Additionally, scores on peer relationship and academic performance were slightly lower in the group with a separation ≥ 3 m, while the scores on depression were slightly higher. However, these differences were not statistically significant (*P* > 0.05).

### Association of parent–child separation with depression, social and academic performance in children during later life

Table 2 displays the result from the investigation on the relationship between various categories of early parent–child separation and depression later in life. After adjusting for covariates, individuals who experienced a separation ≥ 3 m exhibited significantly higher scores at the 50th quantile level (Adjusted *B* = 0.46, 95% CI 0.02 to 0.91, *P* = 0.042) and 75th quantile level (Adjusted *B* = 0.58, 95% CI 0.08 to 1.08, *P* = 0.024) of depression in later life, compared to children without separation experience. No significant difference was observed at the 25th quantile level of depression scores. In contrast, for children who separated from parents for less than 3 months, no significant correlation was identified, regardless of the adjusted model.

**Table 2:** Quantile regression of the association between parent–child separation and depression in children during later life

Variables	Different quantile levels of depression								
	q25			q50			q75		
Parent–child separation	Adj. *B*	95% CI	*P*	Adj. *B*	95% CI	*P*	Adj. *B*	95% CI	*P*
No separation	Ref			Ref			Ref		
Separation < 3 m	− 0.37	(− 0.83, 0.09)	0.115	− 0.47	(− 1.1, 0.26)	0.206	0.004	(− 0.57, 0.58)	0.988
Separation ≥ 3 m	0.32	(− 0.05, 0.68)	0.089	0.46	(0.02, 0.91)	**0.042**	0.58	(0.08, 1.08)	**0.024**
Pseudo R^2^	0.003			0.014			0.031		
N	2817								

Adjusted models: controlled for gender, age in 2010, residence, ethnicity, BMI, paternal and maternal age in 2010, paternal and maternal educational level, family size in 2018 and net household income per capita in 2018.'q25','q50' and'q75' stand for the quantiles at the 25th, 50th, and 75th percentiles, respectively. The CES-D8 scores at q25, q50, and q75 were 2, 4, and 7, respectively. *P* < 0.05 was considered statistically significant

Adj. *B*, Adjusted regression coefficient. CI, Confidence Interval

The study then explored the relationship between various categories of parent–child separation and social and academic performance later in life. First the unadjusted model (Table S1), and then in the fully adjusted model (Table 3). Children who experienced a separation ≥ 3 m scored significantly lower on the parent–child relationship (Adjusted *B* = − 0.14, 95% CI − 0.27 to − 0.02, *P* = 0.027) and peer relationship (Adjusted *B* = − 0.28, 95% CI − 0.50 to − 0.05, *P* = 0.015), compared to the no-separation group. No significant difference was observed in academic performance scores after adjusting for covariates, and no significant correlation was identified in children experienced parent–child separation for less than 3 months.

**Table 3:** Multivariate linear regression of the association of parent–child separation on social and academic performance in children during later life

Variables	Parent–child relationship			Peer relationship			Academic performance		
Parent–child separation	Adj. *B*	95% CI	*P*	Adj. *B*	95% CI	*P*	Adj. *B*	95% CI	*P*
No separation	Ref			Ref			Ref		
Separation < 3 m	0.003	(− 0.13, 0.14)	0.967	0.04	(− 0.20, 0.27)	0.752	0.10	(− 0.03, 0.23)	0.120
Separation ≥ 3 m	−0.14	(−0.27, −0.02)	**0.027**	− 0.28	(− 0.50, − 0.05)	**0.015**	− 0.03	(− 0.15, 0.09)	0.583
R^2^	0.022			0.010			0.010		
F	6.24			2.05			1.23		
N	3428			2817			2022		

Models were adjusted for gender, age in 2010, residence, ethnicity, BMI, paternal and maternal age in 2010, paternal and maternal educational level, family size in 2018. For peer relationship, net household income per capita in 2018 was additionally adjusted; for academic performance, net household income per capita in 2018, education stage in 2018, school location and academic stress were additionally adjusted. *P* < 0.05 was considered statistically significant

### Relationship between parent–child separation and depression according to gender, age, residence, and maternal educational level

**Fig. 2:**
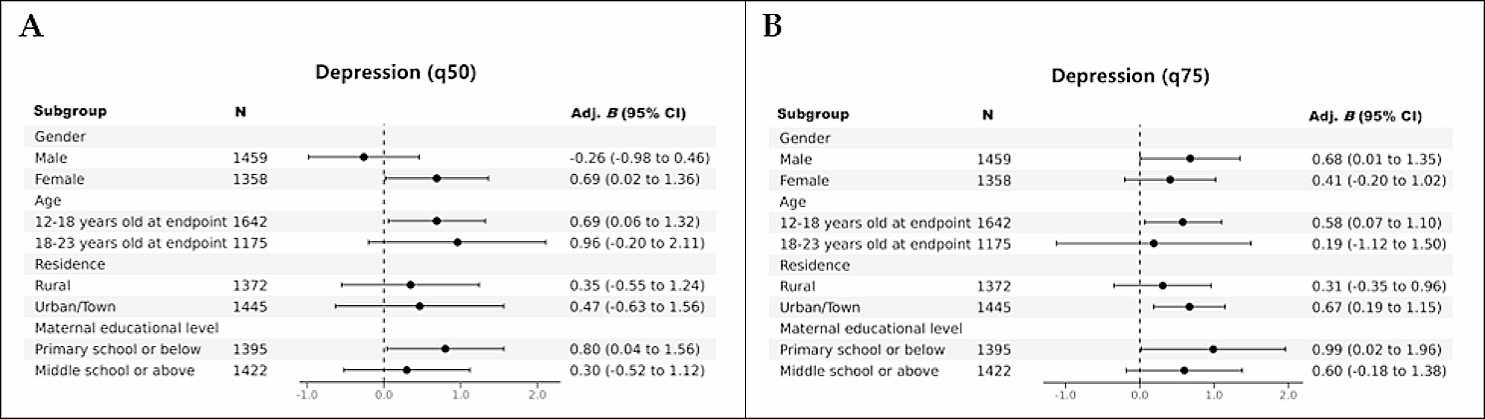
Quantile regression of the association between parent–child separation ≥ 3 months (compared to no parent–child separation group) and depression in children during later life among different subgroups. **A** The 50-quantile level of depression. **B** The 75-quantile level of depression. Models were adjusted for gender, age in 2010, residence, ethnicity, BMI, paternal and maternal age in 2010, paternal and maternal educational level, family size in 2018 and net household income per capita in 2018. The CES-D8 scores at q50 and q75 were 4 and 7, respectively

As presented in Table S2 and Fig. 2A, females (Adjusted *B* = 0.69, 95% CI 0.02 to 1.36, *P* = 0.043), younger age groups (12–18 years at the follow-up endpoint) (Adjusted *B* = 0.69, 95% CI 0.06 to 1.32, *P* = 0.032), children whose mothers' educational level was primary school or below (Adjusted *B* = 0.80, 95% CI 0.04 to 1.56, *P* = 0.038), and children who experienced a separation ≥ 3 m exhibited higher scores at the 50th quantile level of depression later in life. Similarly, at the 75th quantile level of depression in later life, males (Adjusted *B* = 0.68, 95% CI 0.01 to 1.35, *P* = 0.048), younger age groups (12–18 years at the follow-up endpoint) (Adjusted *B* = 0.58, 95% CI 0.07 to 1.10, *P* = 0.027), individuals residing in urban or town areas (Adjusted *B* = 0.67, 95% CI 0.19 to 1.15, *P* = 0.007), children whose mothers' educational level was primary school or below (Adjusted *B* = 0.99, 95% CI 0.02 to 1.96, *P* = 0.046), and children with a separation ≥ 3 m showed higher scores on depression (Table S2 and Fig. 2B).

### Relationship between parent–child separation and social and academic performance according to gender, age, residence, and maternal educational level

**Fig. 3:**
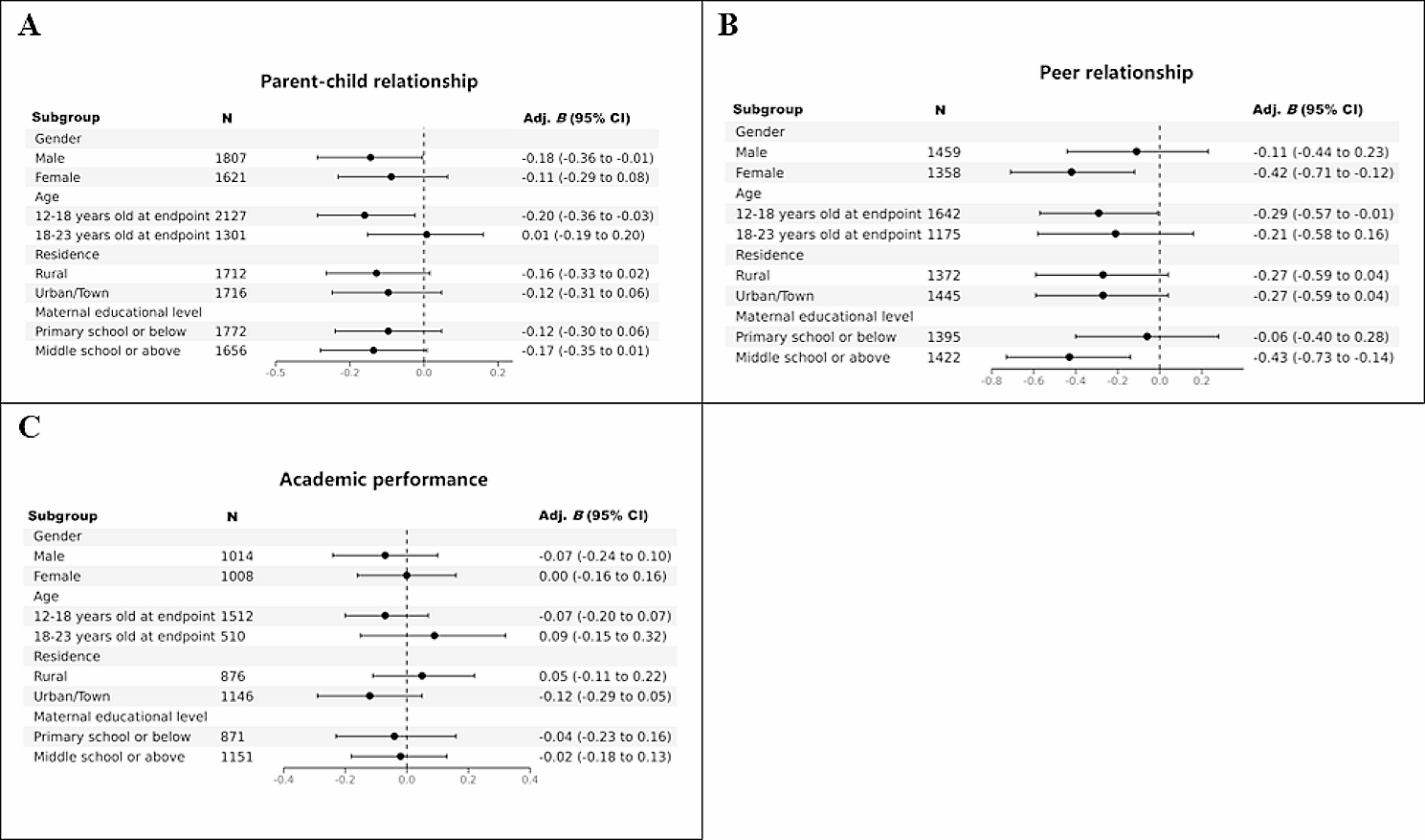
Multivariate linear regression of the association between parent–child separation ≥ 3 months (compared to no parent–child separation group) and social and academic performance in children during later life among different subgroups. The association between parent–child separation ≥ 3 months and parent–child relationship (**A**), peer relationship (**B**), and academic performance (**C**). Models were adjusted for gender, age in 2010, residence, ethnicity, BMI, paternal and maternal age in 2010, paternal and maternal educational level, family size in 2018. For peer relationship, net household income per capita in 2018 was additionally adjusted; for academic performance, net household income per capita in 2018, education stage in 2018, school location and academic stress were additionally adjusted

As illustrated in Table S3 and Fig. 3A, males (Adjusted *B* = − 0.18, 95% CI − 0.36 to − 0.01, *P* = 0.044), younger age groups (12–18 years at the follow-up endpoint) (Adjusted *B* = − 0.20, 95% CI − 0.36 to − 0.03, *P* = 0.018), and those who experienced a separation ≥ 3 m scored lower on parent–child relationship later in life. Similarly, as shown in Table S4 and Fig. 3B, females (Adjusted *B* = − 0.42, 95% CI − 0.71 to − 0.12, *P* = 0.006), younger age groups (12–18 years at the follow-up endpoint) (Adjusted *B* = − 0.29, 95% CI − 0.57 to − 0.01, *P* = 0.045), children whose mothers' educational level was middle school or above (Adjusted *B* = − 0.43, 95% CI − 0.73 to − 0.14, *P* = 0.004), and those who experienced a separation ≥ 3 m scored lower on peer relationship later in life. However, the relationship between parent–child separation and later academic performance was not significant across different genders, ages, residential areas, and maternal education levels (Table S5 and Fig. 3C).

### Association between the separation duration and depression as well as social and academic performance in children during later life

To investigate the "dose–response" relationship between the duration of separation and depression, as well as the relationship between separation duration and social and academic performance, an RCS model was utilized and presented in Figures S1 and S2. We further conducted regression analysis incorporating the square term of separation duration, which revealed a significant positive correlation between the square of separation duration and depression scores (Table S6). Additionally, an RCS model controlled for covariates was shown in Figure S3. This finding suggests a "dose–response" relationship between separation duration and depression. At the turning point of 3 months, the curve changed its direction. The data was divided into two segments based on the cut-off value at 3 months, and segmented linear regression showed a significant positive association between separation duration and the depression score in children who experienced a separation ≥ 3 m (Adjusted *B* = 0.06, 95% CI 0.01 to 0.11, *P* = 0.013). For children with a separation duration less than 3 months, there was an inverse association between separation duration and depression (Adjusted *B* = − 0.82, 95% CI − 1.72 to 0.07, *P* = 0.071), but it did not reach significance (Table 4).

**Table 4:** Segmented linear regression of the association between separation duration and depression in children during later life

Variables	Separation duration (< 3 m)			Separation duration (≥ 3 m)		
	Adj. *B*	95% CI	*P*	Adj. *B*	95% CI	*P*
Separation duration, m	− 0.82	(− 1.72, 0.07)	0.071	0.06	(0.01, 0.11)	**0.013**
R^2^	0.099			0.079		
F	2.11			1.86		
N	244			274		

Models were adjusted for gender, age in 2010, residence, ethnicity, BMI, paternal and maternal age in 2010, paternal and maternal educational level, family size in 2018 and net household income per capita in 2018. *P* < 0.05 was considered statistically significant

The curves displayed a linear pattern for separation duration and social and academic performance scores, as shown in Figure S2. Therefore, multivariate linear regression was used to explore these associations. The results showed a significant inverse association between separation duration and parent–child relationship (Adjusted *B* = − 0.02, 95% CI − 0.03 to − 0.01, *P* = 0.039), peer relationship (Adjusted *B* = − 0.02, 95% CI − 0.04 to − 0.01, *P* = 0.046), and academic performance (Adjusted *B* = − 0.01, 95% CI − 0.02 to − 0.001, *P* = 0.035) (Table 5).

**Table 5:** Multivariate linear regression of the association of the separation duration on social and academic performance in children during later life

Variables	Parent–child relationship			Peer relationship			Academic performance		
	Adj. *B*	95% CI	*P*	Adj. *B*	95% CI	*P*	Adj. *B*	95% CI	*P*
Separation duration, m	− 0.02	− 0.03, − 0.01	**0.039**	− 0.02	− 0.04, − 0.01	**0.046**	− 0.01	− 0.02, − 0.001	**0.035**
R^2^	0.050			**0.033**			0.026		
F	2.212			1.434			0.98		
N	518			518			495		

Models were adjusted for gender, age in 2010, residence, ethnicity, BMI, paternal and maternal age in 2010, paternal and maternal educational level, family size in 2018 and net household income per capita in 2018. For academic performance, due to insufficient valid samples, net household income per capita in 2018 was not adjusted. Education stage in 2018 and school location were additionally adjusted. *P* < 0.05 was considered statistically significant

### Robustness analysis

After applying multiple imputations to address missing data in academic performance and covariates, we conducted additional statistical analyses to examine the robustness of our findings. The results are presented in Tables S7–S9 of the supplementary materials, which indicate that the main findings remained robust even after considering the effects of multiple imputations on missing data.

## Discussion

This study identified three main findings. Firstly, children who experienced separation from their parents for 3 months or longer had a higher risk of moderate to severe levels of depression and poorer parent–child and peer relationships later in life compared to children without such separation. Secondly, the association between parent–child separation and depression, social and academic performance, varied based on factors such as gender, age groups, residence, and maternal education levels. Lastly, the study discovered positive correlation between separation duration and depression among children who were separated from their parents for 3 months or longer, and negative correlations between separation duration and social relationship, and academic performance in children who experienced separation.

Existing research on the association between parent–child separation and child development is limited to cross-sectional studies [43, 44] and short-term longitudinal tracking [45]. The current study examined the long-term effects of early separation on children and their subsequent health outcomes. The findings support the first hypothesis that prolonged parent–child separation in early life worsens children’s later depression status, social and academic performance. A previous longitudinal study by Coffino [46] showed no association between parent–child separation during early childhood (0–4 years) and adult depression at age 26, while separation between ages 5 and 8 significantly predicted adult depression at age 26. It is worth noting that in the present study, for children who experienced early parent–child separation for 3 months or longer, over 95% of their parents were married, and they were younger, with larger family size and poorer economic conditions, indicating labor migration is a potential cause of early parent–child separation. Conversely, Coffino's study attributed separation primarily to single mothers raising children alone due to premarital pregnancies [46]. This discrepancy in separation causes between studies may explain the observed outcome differences. Consistent with our findings, Yang et al. [21] also found that for children younger than 3 years old, separation from both parents significantly increased their risk of later depression. Additionally, Zhao et al. [19] examined the effects of separation from both parents and separation from one parent on children's later depression and behavior. They found that separation from both parents was associated with higher levels of depression and internalizing behaviors compared to separation from one parent, which indicate a more severe impact of separation from both parents on children's later mental health. Liu et al. [47] suggested that children who experienced separation from their parents at a younger age had a higher risk of developing depression during adolescence. These research findings further emphasize the potential impact of early separation from parents on children's psychological well-being. Furthermore, the present study also found that parent–child separation for 3 months or longer before the age of 3 reduces the quality of parent–child and peer relationships later in life. Although existing evidence cannot be directly compared to our findings, potential psychological mechanisms may be related to the experience of emotional deprivation, lack of security, and difficulties in forming self-identity following separation [48, 49]. A prospective cohort study found that greater parental involvement during caregiving for children aged 0–7 years old is associated with a reduced risk of depression at 18 years old [50]. Conversely, inadequate parental care is linked to increased vulnerability to emotional and behavioral problems, as well as difficulties in peer relationships [51].

Further analysis of the impact of parent–child separation, after taking factors such as gender, age group, residence, and maternal education level into account, confirmed the second hypothesis of the study. Females who experienced separation for 3 months or longer were more likely to exhibit moderate levels of depression (as indicated by CES-D8 scores of 4 or higher) and poorer peer relationship. Conversely, males who experienced separation for 3 months or longer exhibited a higher risk of developing severe levels of depression (as indicated by CES-D8 scores of 7 or higher) and impaired parent–child relationship. These differences may reflect variations in coping mechanisms and adaptation processes between males and females in response to parent–child separation. While existing evidence suggests a higher susceptibility to depression among females [52–54], this study revealed that prolonged parent–child separation may pose a greater risk of severe levels of depression for males. This may be attributed to societal expectations and gender roles, as males are often expected to assume more responsibility and independence [55]. Prolonged parent–child separation impacts their self-identity and emotional well-being [56], leading to increased susceptibility to depression caused by social pressures. Additionally, age differences were observed in the study. Parent–child separation was positively associated with the severity of depression and negatively associated with parent–child and peer relationships in the 12–18 age group, while no associations were found in the age group older than 18. Erikson's theory of development stages may explain these differences, as adolescents rely more on parent–child and peer relationships to fulfill their senses of identity and emotional needs [57–59], making them more sensitive to the impact of early parent–child separation. In contrast, young adults rely more on their own abilities and social support networks, hence, they could reduce their sensitivity to the adverse effects of early parent–child separation. Regarding different residence, consistent with previous study [21], the results showed that children living in urban/town areas were more likely to exhibit more severe depression if they experienced parent–child separation for more than 3 months. This finding contradicts Zhao's findings, as they found that rural children who experienced parent–child separation were more prone to depression during adolescence [19]. This discrepancy may be attributed to the difference in the age period when children experienced parent–child separation. Contrary to existing research [19, 20], the present study found no significant differences in the rates of parent–child separation between urban and rural areas. This unexpected finding challenges the initial assumption that rural areas would have a higher prevalence of separation. Our analysis indicates that this result may be attributed to the definition and measurement methods we employed for assessing separation. Regardless, the findings highlight the importance of addressing the mental health of children from both rural and urban/town areas who are left behind. Additionally, we found that children with mothers who have lower educational levels were more likely to experience moderate to severe levels of depression after experiencing early separation for 3 months or longer. This suggests that mothers with lower educational levels lack the educational resource and are unable to provide sufficient supports to their children.

Subgroup analysis of individuals who experienced parent–child separation revealed that only children with separation lasting 3 months or longer showed a positive correlation between separation duration and later depression. However, short-term separations of less than 3 months did not exhibit this association. Although the result was not entirely consistent with the third research hypothesis, this phenomenon may suggest that the detrimental impact of parent–child separation on future health outcomes largely depend on the "dose–response" relationship. From the perspective of life history strategies in evolutionary ecology, early experiences have a U-shaped effect on children's physiological stress system [60]. Temporary parent–child separation is believed to trigger more intermittent and ultimately manageable physiological and psychological stress responses. These responses, sometimes referred to as "strengthening" [61] or "stress inoculation" [62], could help buffer the organism against subsequent environmental stressors through moderate activation and recovery of these systems. However, prolonged separation may lead to excessive activation of systems, ultimately inducing maladaptive compensatory adaptations [60, 63, 64]. This may explain why only longer durations of separation showed a positive correlation with later depression, while short-term separations did not exhibit this association. Furthermore, for children who experienced early separation, there was a linear negative correlation between separation duration and social and academic performance, suggesting that longer separation duration reduces the quality of parent–child relationship, peer relationship, and academic performance later in life. Previous research has indicated that inadequate parental care increases children's vulnerability to emotional and behavioral problems, as well as difficulties in forming better peer relationships [51]. This further supports our research findings that parent–child separation has long-term effects on children's social and health outcomes.

Given the findings of this study, actionable intervention strategies are proposed. Firstly, in infancy and early childhood, efforts should be made to minimize separations lasting longer than 3 months between parents and children. Prioritize creating supportive environments and policies to keep children aged 3 and below with their parents. Secondly, tailored interventions should be designed for high-risk populations, such as males, adolescents, urban residents, and individuals with lower maternal education levels. Provide additional support and resources to meet their specific needs. Thirdly, offer academic support to children and adolescents who have experienced prolonged separations, such as targeted educational programs, counseling, and additional resources to mitigate potential impacts on their academic development. Implementing these strategies aims to reduce the risk of future mental health issues and promote the well-being of children and adolescents who have experienced early parent–child separation.

### Limitations

Despite being an 8-year longitudinal study based on a nationally representative population, with a well-designed and rigorously controlled cohort [30], this study has some limitations that need to be acknowledged. First, during the 8-year follow-up period, a total of 43% of the data gradually became lost to follow-up. A comparison of baseline demographic characteristics between participants included in the analysis and those lost to follow-up revealed that a higher proportion of the latter group experienced parent–child separation before age 3 (10.4% vs. 14% in the lost to follow-up group). The lost to follow-up group also had older parents and higher levels of parental education (Table S10). These factors may have introduced bias into the results. Second, this study examined the impact of early childhood dual-parent separation on later life health outcomes, contrasting with a no-separation cohort. However, single-parent separation data that could be present in the no-separation cohort were not excluded due to data limitations, potentially cause bias. Third, the specific reasons for parent–child separation for the children at or before the age of 3 could not be determined in the available database. Nevertheless, an analysis of the 2010 baseline data on parents not living at home during 2010 indicated that certain causes of separation, such as parental divorce (23 cases, 3.3%) and father's incarceration (2 cases, 0.3%), may introduce some degree of bias in the results. However, the low prevalence of these causes helps minimize the potential bias. Fourth, despite controlling for as many confounding factors as possible and conducting various subgroup analyses, other potential confounders such as early childhood outcome variables, caregiver parenting styles and emotional support may still have an impact on the results.

In future research, it would be valuable to account for the nested structure, such as multilevel modeling, and to explore the potential benefits of using sampling weights or propensity scores to enhance the representativeness of the findings. Additionally, further exploration is needed to understand the potential mechanisms underlying the impact of early parent–child separation on children's later health outcomes, as well as to investigate whether positive caregiver support and social support can mitigate negative health outcomes.

## Conclusion

The present study provides evidence that parent–child separation lasting 3 months or longer during infancy and early childhood significantly increase the risk of depression and impair social and academic performance during adolescence and young adulthood. This effect is particularly pronounced among males, adolescents, urban dwellers, and individuals with less educated mothers. Furthermore, the duration of separation is negatively associated with both parent–child and peer relationships, as well as academic performance. Importantly, a "dose–response" relationship was observed between the severity of depression and the duration of separation, highlighting the cumulative impact of prolonged separation. These findings highlight the critical importance of minimizing separations to less than 3 months for children under the age of 3 to mitigate the risk of future mental health issues. Moreover, the results underscore the crucial role of early parental care and emphasize the necessity of targeted interventions for high-risk populations.

### Electronic supplementary material

Below is the link to the electronic supplementary material.

Supplementary Material 1.

Supplementary Material 2.

## Data Availability

The datasets employed in this study are accessible via the China Family Panel Studies (CFPS) website at http://www.isss.pku.edu.cn/cfps/en/.

## Abbreviation

CFPS: China Family Panel Studies
CES-D: Center for epidemiologic studies depression scale
BMI: Body mass index
SD: Standard Deviation
CI: Confidence intervals
RCS: Restricted cubic spline
