# Supplementary material for: Association of early parent–child separation with depression, social and academic performance in adolescence and early adulthood: a prospective cohort study
Source: Child Adolesc Psychiatry Ment Health. 2024 Jun 26;18:78. doi: 10.1186/s13034-024-00769-1 (PMC11210141; doi:10.1186/s13034-024-00769-1)
Supplement: Supplementary file 1 — Supplementary Material 1. [file 13034_2024_769_MOESM1_ESM.docx]

Association of early parent-child separation with depression, social and academic performance in adolescence and early adulthood: A Prospective Cohort Study

Honghua Li^1, 2^, Kai Liu^1^, Junsong Fei^1^, Tongshuang Yuan^1^, Songli Mei ^1,^ *

^1^ Department of Social Medicine and Health Management, School of Public Health, Jilin University, Changchun 130021, Jilin Province, China.

^2^ Department of Developmental and Behavioral Pediatrics, Children's Medical Center, The First Hospital of Jilin University, Changchun 130021, Jilin Province, China.

***Corresponding author**:

Songli Mei, PhD

Department of Social Medicine and Health Management, School of Public Health, Jilin University, No. 1163 Xinmin Street, Changchun 130021, Jilin Province, China.

Tel: +8613104303560; Fax: +8643185619454

E-mail: [meisongli@sina.com](mailto:meisongli@sina.com)

**Supplementary Materials:**

**Figure S1** Association between duration of parent-child separation and depression. RCS model with knots at 5th, 35th, 65th, and 90th percentiles. Y-axis represents the unadjusted *B* for any value of separation duration compared to individuals with 3 months of separation duration.

**Figure S2** Association between duration of parent-child separation and parent-child relationship (A), peer relationship (B), and academic performance (C). RCS models with knots at 10th, 50th, and 90th percentiles. Y-axis represents unadjusted *B* for any value of separation duration compared to individuals with 1.2 months of separation duration. All the linear regression was unadjusted.

**Figure S3** Association between separation duration and depression with the RCS function. Model with 4 knots located at 5th, 35th, 65th and 95th percentiles. Y-axis represents the adjusted B to present depression for any value of separation duration compared to individuals with reference value (3 months) of separation duration.

**Table S1** Multivariate linear regression (without controlling for covariates) of the association of parent-child separation on social and academic performance in children during later life

**Table S2** Quantile regression of the association between parent-child separation and depression in children during later life among different subgroups

**Table S3** Multivariate linear regression of the association between parent-child separation and parent-child relationship in children during later life among different subgroups

**Table S4** Multivariate linear regression of the association between parent-child separation and peer relationship in children during later life among different subgroups

**Table S5** Multivariate linear regression of the association between parent-child separation and academic performance in children during later life among different subgroups

**Table S6** Regression analysis of the square of separation duration and depression

**Table S7** Quantile regression of the association between parent-child separation and depression in children during later life after multiple imputation

**Table S8** Multivariate linear regression of the association of parent-child separation on social and academic performance in children during later life after multiple imputation

**Table S9** Multivariate linear regression of the association of the separation duration on social and academic performance in children during later life after multiple imputation

**Table S10** Baseline characteristics in the follow-up and lost to follow-up participants


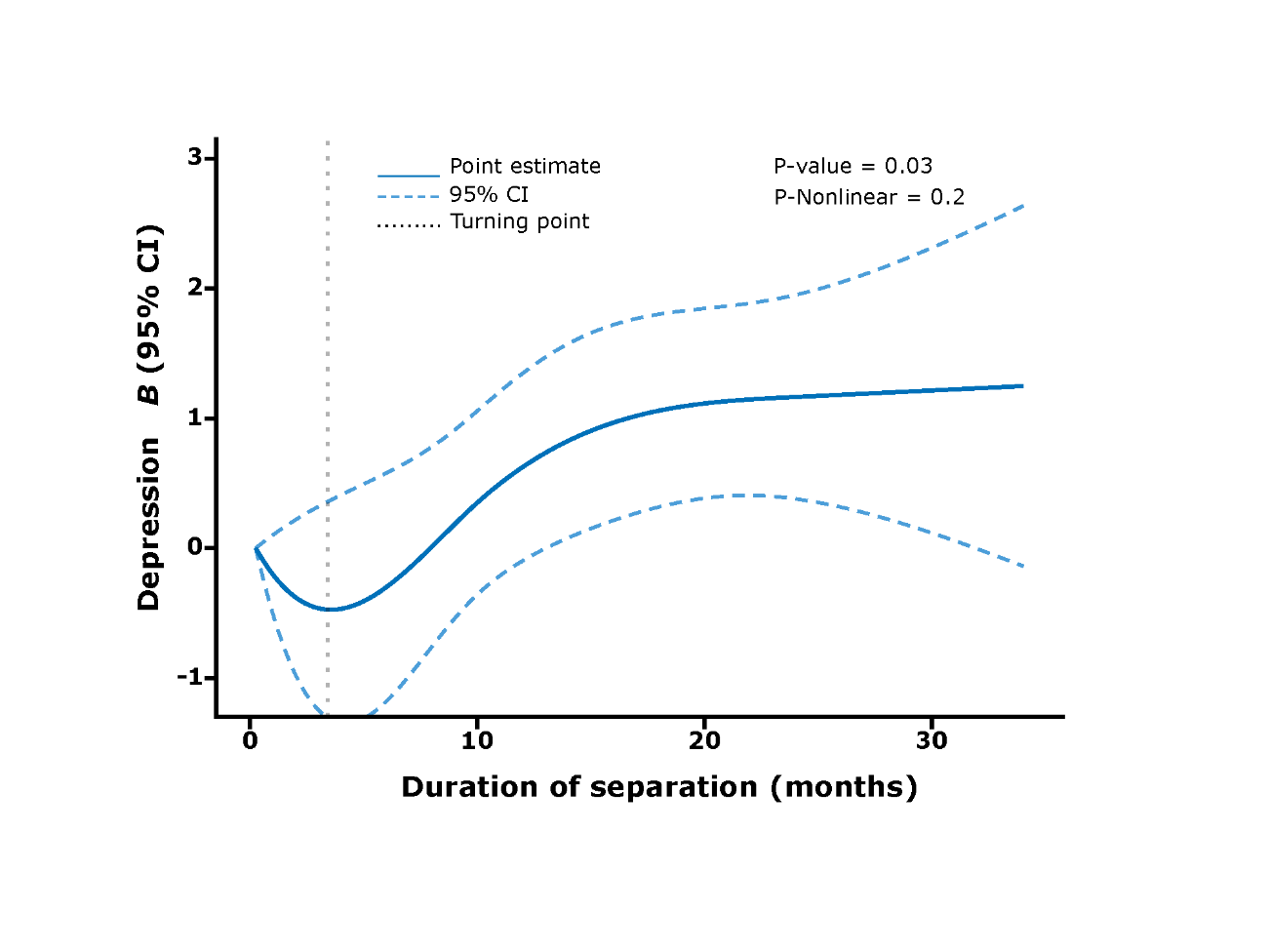


**Figure S1** Association between duration of parent-child separation and depression. RCS model with knots at 5th, 35th, 65th, and 90th percentiles. Y-axis represents the unadjusted *B* for any value of separation duration compared to individuals with 3 months of separation duration.

| **A**  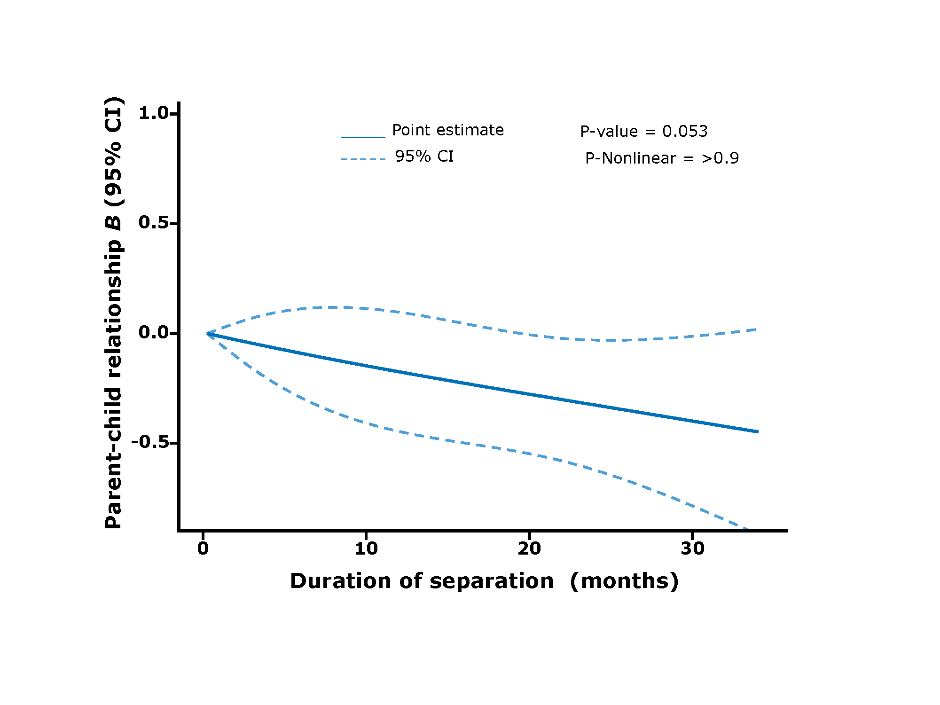 | **B**  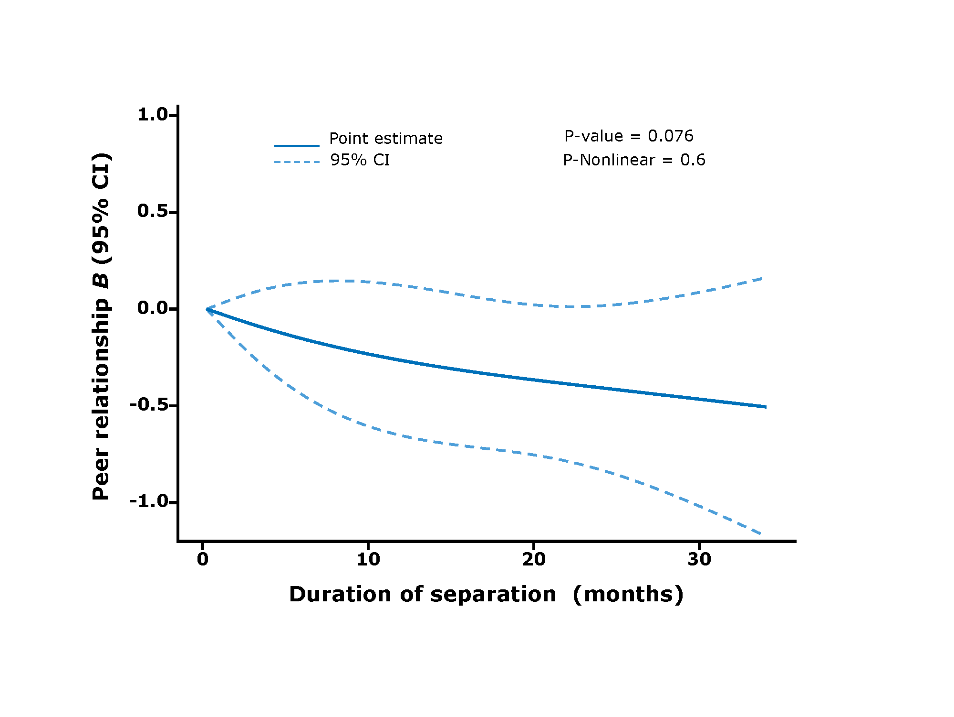 |
| --- | --- |
| **C** 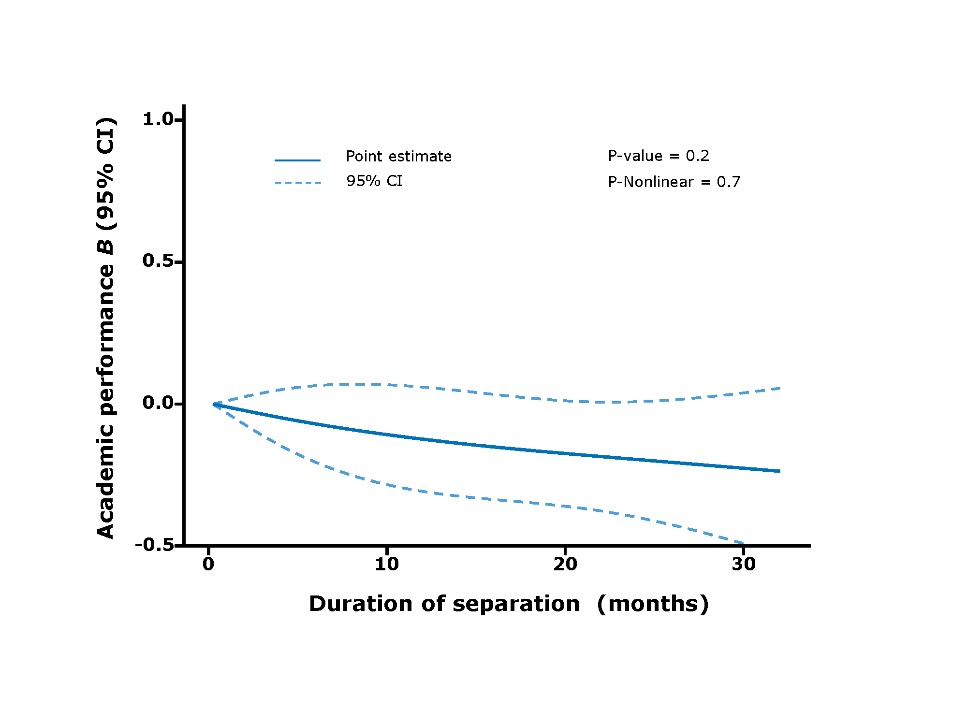 |  |

**Figure S2** Association between duration of parent-child separation and parent-child relationship (A), peer relationship (B), and academic performance (C). RCS models with knots at 10th, 50th, and 90th percentiles. Y-axis represents unadjusted *B* for any value of separation duration compared to individuals with 1.2 months of separation duration. All the linear regression was unadjusted.


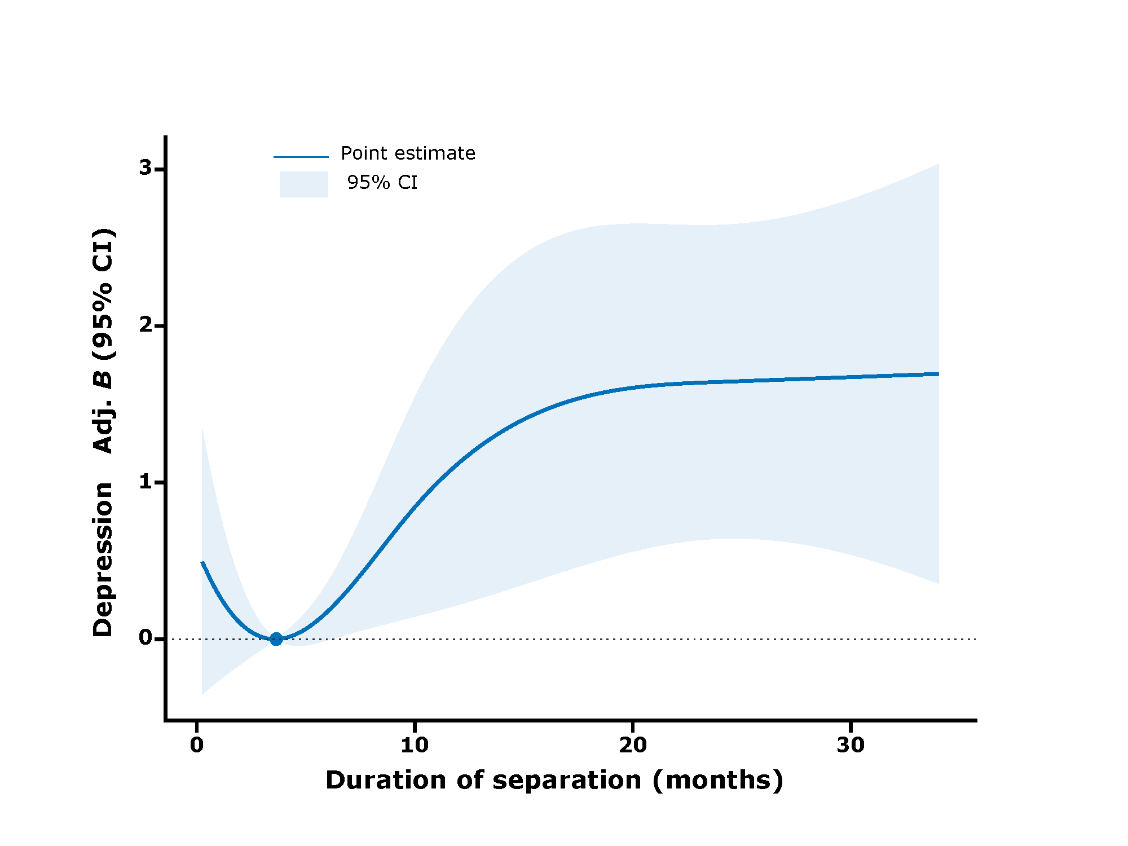


**Figure S3** Association between separation duration and depression with the RCS function. Model with 4 knots located at 5th, 35th, 65th and 95th percentiles. Y-axis represents the adjusted *B* to present

depression for any value of separation duration compared to individuals with reference value (3 months) of separation duration. Model was adjusted for gender, age in 2010, residence, ethnicity, BMI, paternal and maternal age in 2010, paternal and maternal educational level, family size in 2018 and net household income per capita in 2018.

**Table S1 Multivariate linear regression (without controlling for covariates) of the association of parent-child separation on social and academic performance in children during later life.**

| **Variables** | **Parent-child relationship** | | **Peer relationship** | | **Academic performance** | |
| --- | --- | --- | --- | --- | --- | --- |
| Parent-child separation | Unadjusted *B*  (95% CI) | *P* | Unadjusted *B*  (95% CI) | *P* | Unadjusted *B*  (95% CI) | *P* |
| No separation | Ref. |  | Ref. |  | Ref. |  |
| Separation < 3m | -0.02 (-0.15, 0.12) | 0.819 | 0.05 (-0.16, 0.26) | 0.641 | 0.05 (-0.07, 0.16) | 0.416 |
| Separation ≥ 3m | **-0.30 (-0.41, -0.18)** | **＜0.001** | -0.17 (-0.36, 0.01) | 0.065 | -0.09 (-0.19, 0.01) | 0.068 |
| R^2^ | 0.006 | | 0.001 | | 0.002 | |
| F | 11.80 | | 1.92 | | 2.19 | |
| N | 3829 | | 3829 | | 2856 | |

*B* = Unstandardized regression coefficients. CI = Confidence Interval.

**Table S2 Quantile regression of the association between parent-child separation and depression in children during later life among different subgroups ^a b^**

| **Subgroup** | **Depression (q50)** | | **Depression (q75)** | |
| --- | --- | --- | --- | --- |
| Gender subgroups | Adjusted *B*  (95% CI) | *P* | Adjusted *B*  (95% CI) | *P* |
| *Male (n=1459)* |  |  |  |  |
| Parent-child separation < 3m | -0.30 (-1.30, 0.69) | 0.550 | 0.07 (-0.59, 0.72) | 0.846 |
| Parent-child separation ≥ 3m | -0.26 (-0.98, 0.46) | 0.473 | **0.68 (0.01, 1.35)** | **0.048** |
| *Female (n=1358)* |  |  |  |  |
| Parent-child separation < 3m | -0.68 (-1.48, 0.12) | 0.094 | -0.04 (-0.94, 0.85) | 0.922 |
| Parent-child separation ≥ 3m | **0.69 (0.02, 1.36)** | **0.043** | 0.41 (-0.20, 1.02) | 0.191 |
| ***Age subgroups*** |  |  |  |  |
| *12-18 years old at endpoint (n=1642)* |  |  |  |  |
| Parent-child separation < 3m | -0.20 (-1.14, 0.75) | 0.685 | 0.30 (-0.39, 0.99) | 0.392 |
| Parent-child separation ≥ 3m | **0.69 (0.06, 1.32)** | **0.032** | **0.58 (0.07, 1.10)** | **0.027** |
| *18-23 years old at endpoint (n=1175)* |  |  |  |  |
| Parent-child separation < 3m | -0.59 (-1.47, 0.28) | 0.185 | -0.14 (-1.59, 1.32) | 0.855 |
| Parent-child separation ≥ 3m | 0.96 (-0.20, 2.11) | 0.106 | 0.19 (-1.12, 1.50) | 0.777 |
| ***Residence subgroups*** |  |  |  |  |
| *Rural (n=1372)* |  |  |  |  |
| Parent-child separation < 3m | -0.44 (-1.48, 0.60) | 0.403 | 0.30 (-0.86, 1.46) | 0.611 |
| Parent-child separation ≥ 3m | 0.35 (-0.55, 1.24) | 0.449 | 0.31 (-0.35, 0.96) | 0.358 |
| *Urban/Town (n=1445)* |  |  |  |  |
| Parent-child separation < 3m | -0.44 (-1.42, 0.54) | 0.378 | 0.54 (-0.46, 1.55) | 0.289 |
| Parent-child separation ≥ 3m | 0.47 (-0.63, 1.56) | 0.406 | **0.67 (0.19, 1.15)** | **0.007** |
| ***Maternal education subgroups*** |  |  |  |  |
| *Primary school or below (n=1395)* |  |  |  |  |
| Parent-child separation < 3m | 0.08 (-0.52, 0.69) | 0.786 | 0.57 (-0.57, 1.71) | 0.326 |
| Parent-child separation ≥ 3m | **0.80 (0.04, 1.56)** | **0.038** | **0.99 (0.02, 1.96)** | **0.046** |
| *Middle school or above (n=1422)* |  |  |  |  |
| Parent-child separation < 3m | **-1.25 (-2.39, -0.12)** | **0.031** | -0.22 (-0.88, 0.44) | 0.516 |
| Parent-child separation ≥ 3m | 0.30 (-0.52, 1.12) | 0.476 | 0.60 (-0.18, 1.38) | 0.133 |

**^a^** Compared to no parent-child separation group. **^b^** Models were adjusted for gender, age in 2010, residence, ethnicity, BMI, paternal and maternal age in 2010, paternal and maternal educational level, family size in 2018 and net household income per capita in 2018. *B* = Unstandardized regression coefficients. CI = Confidence Interval. The CES-D8 scores at q50 and q75 were 4 and 7, respectively.

**Table S3 Multivariate linear regression of the association between parent-child separation and** **parent-child relationship in children during later life among different subgroups** **^a b^**

| **Subgroup** | **Unadjusted *B***  **(95% CI)** | ***P*** | **Adjusted *B***  **(95% CI)** | ***P*** |
| --- | --- | --- | --- | --- |
| ***Gender subgroups*** |  |  |  |  |
| *Male* | *n=2018* |  | *n=1807* |  |
| Parent-child separation < 3m | -0.03 (-0.21, 0.16) | 0.760 | -0.01 (-0.19, 0.17) | 0.915 |
| Parent-child separation ≥ 3m | **-0.32 (-0.48, -0.15)** | **＜0.001** | **-0.18 (-0.36, -0.01)** | **0.044** |
| *Female* | *n=1811* |  | *n=1621* |  |
| Parent-child separation < 3m | 0.01 (-0.19, 0.20) | 0.954 | 0.01 (-0.19, 0.22) | 0.884 |
| Parent-child separation ≥ 3m | **-0.26 (-0.43, -0.09)** | **0.003** | -0.11 (-0.29, 0.08) | 0.258 |
| ***Age subgroups*** |  |  |  |  |
| *12-18 years old at endpoint* | *n=2391* |  | *n=2127* |  |
| Parent-child separation < 3m | -0.04 (-0.22, 0.14) | 0.666 | -0.03 (-0.21, 0.16) | 0.788 |
| Parent-child separation ≥ 3m | -0.35 (-0.51, -0.20) | **＜0.001** | **-0.20 (-0.36, -0.03)** | **0.018** |
| *18-23 years old at endpoint* | *n=1438* |  | *n=1301* |  |
| Parent-child separation < 3m | 0.05 (-0.13, 0.22) | 0.615 | 0.03 (-0.14, 0.21) | 0.670 |
| Parent-child separation ≥ 3m | -0.01 (-0.20, 0.18) | 0.908 | 0.01 (-0.19, 0.20) | 0.950 |
| ***Residence subgroups*** |  |  |  |  |
| *Rural* | *n=1965* |  | *n=1712* |  |
| Parent-child separation < 3m | 0.04 (-0.15, 0.23) | 0.692 | 0.07 (-0.12, 0.27) | 0.452 |
| Parent-child separation ≥ 3m | -0.31 (-0.48, -0.15) | **＜0.001** | -0.16 (-0.33, 0.02) | 0.078 |
| *Urban/Town* | *n=1864* |  | *n=1716* |  |
| Parent-child separation < 3m | -0.07 (-0.25, 0.12) | 0.482 | -0.07 (-0.26, 0.12) | 0.499 |
| Parent-child separation ≥ 3m | **-0.27 (-0.45, -0.10)** | **0.002** | -0.12 (-0.31, 0.06) | 0.190 |
| ***Maternal education subgroups*** |  |  |  |  |
| *Primary school or below* | *n=2012* |  | *n=1772* |  |
| Parent-child separation < 3m | -0.004 (-0.18, 0.18) | 0.967 | 0.003 (-0.18, 0.18) | 0.978 |
| Parent-child separation ≥ 3m | **-0.23 (-0.40, -0.06)** | **0.008** | -0.12 (-0.30, 0.06) | 0.177 |
| *Middle school or above* | *n=1741* |  | *n=1656* |  |
| Parent-child separation < 3m | -0.22 (-0.22, 0.18) | 0.860 | 0.001 (-0.20, 0.20) | 0.992 |
| Parent-child separation ≥ 3m | **-0.27 (-0.45, -0.10)** | **0.002** | -0.17 (-0.35, 0.01) | 0.063 |

**^a^** Compared to no parent-child separation group. **^b^** Adjusted models controlled for gender, age in 2010, residence, ethnicity, BMI, paternal and maternal age in 2010, paternal and maternal educational level, family size in 2018. *B* = Unstandardized regression coefficients. CI = Confidence Interval.

**Table S4 Multivariate linear regression of the association between parent-child separation and peer relationship in children during later life among different subgroups ^a b^**

| **Subgroup** | **Unadjusted *B***  **(95% CI)** | ***P*** | **Adjusted *B***  **(95% CI)** | ***P*** |
| --- | --- | --- | --- | --- |
| ***Gender subgroups*** |  |  |  |  |
| *Male* | *n=2018* |  | *n=1459* |  |
| Parent-child separation < 3m | -0.08 (-0.39, 0.22) | 0.589 | -0.04 (-0.38, 0.31) | 0.839 |
| Parent-child separation ≥ 3m | 0.05 (-0.23, 0.32) | 0.730 | -0.11 (-0.44, 0.23) | 0.535 |
| *Female* | *n=1811* |  | *n=1358* |  |
| Parent-child separation < 3m | 0.19 (-0.09, 0.48) | 0.180 | 0.10 (-0.22, 0.43) | 0.524 |
| Parent-child separation ≥ 3m | -0.36 (-0.61, -0.12) | 0.004 | -0.42 (-0.71, -0.12) | 0.006 |
| ***Age subgroups*** |  |  |  |  |
| *12-18 years old at endpoint* | *n=2391* |  | *n=1642* |  |
| Parent-child separation < 3m | -0.02 (-0.29, 0.26) | 0.893 | 0.02 (-0.30, 0.34) | 0.895 |
| Parent-child separation ≥ 3m | -0.21 (-0.44, 0.01) | 0.067 | -0.29 (-0.57, -0.01) | 0.045 |
| *18-23 years old at endpoint* | *n=1438* |  | *n=1175* |  |
| Parent-child separation < 3m | 0.16 (-0.15, 0.48) | 0.309 | 0.05 (-0.29, 0.39) | 0.779 |
| Parent-child separation ≥ 3m | -0.11 (-0.44, 0.22) | 0.513 | -0.21 (-0.58, 0.16) | 0.267 |
| ***Residence subgroups*** |  |  |  |  |
| *Rural* | *n=1965* |  | *n=1372* |  |
| Parent-child separation < 3m | 0.28 (-0.02, 0.58) | 0.064 | 0.28 (-0.06, 0.63) | 0.110 |
| Parent-child separation ≥ 3m | -0.16 (-0.41, 0.10) | 0.224 | -0.27 (-0.59, 0.04) | 0.091 |
| *Urban/Town* | *n=1864* |  | *n=1445* |  |
| Parent-child separation < 3m | -0.18 (-0.47, 0.11) | 0.231 | -0.19 (-0.51, 0.12) | 0.235 |
| Parent-child separation ≥ 3m | -0.19 (-0.46, 0.08) | 0.169 | -0.27 (-0.59, 0.04) | 0.091 |
| ***Maternal education subgroups*** |  |  |  |  |
| *Primary school or below* | *n=2012* |  | *n=1395* |  |
| Parent-child separation < 3m | 0.04 (-0.25, 0.33) | 0.793 | 0.05 (-0.29, 0.39) | 0.778 |
| Parent-child separation ≥ 3m | -0.07 ( -0.35, 0.20) | 0.605 | -0.06 (-0.40, 0.28) | 0.712 |
| *Middle school or above* | *n=1741* |  | *n=1422* |  |
| Parent-child separation < 3m | 0.08 (-0.23, 0.38) | 0.627 | 0.01 (-0.31, 0.33) | 0.943 |
| Parent-child separation ≥ 3m | -0.32 (-0.58, -0.06) | 0.017 | -0.43 (-0.73, -0.14) | 0.004 |

**^a^** Compared to no parent-child separation group. **^b^** The adjusted models controlled for gender, age in 2010, residence, ethnicity, BMI, paternal and maternal age in 2010, paternal and maternal educational level, family size in 2018 and net household income per capita in 2018. *B* = Unstandardized regression coefficients. CI = Confidence Interval.

**Table S5 Multivariate linear regression of the association between parent-child separation and academic performance in children during later life among different subgroups** **^a, b^**

| **Subgroup** | **Unadjusted *B***  **(95% CI)** | ***P*** | **Adjusted *B***  **(95% CI)** | ***P*** |
| --- | --- | --- | --- | --- |
| ***Gender subgroups*** |  |  |  |  |
| *Male* | *n=1471* |  | *n=1014* |  |
| Parent-child separation < 3m | 0.02 (-0.14, 0.19) | 0.789 | 0.09 (-0.09, 0.27) | 0.342 |
| Parent-child separation ≥ 3m | **-0.14 (-0.29, -0.002)** | **0.047** | -0.07 (-0.24, 0.10) | 0.399 |
| *Female* | *n=1385* |  | *n=1008* |  |
| Parent-child separation < 3m | 0.07 (-0.08, 0.23) | 0.358 | 0.09 (-0.08, 0.27) | 0.293 |
| Parent-child separation ≥ 3m | -0.04 (-0.17, 0.09) | 0.571 | 0.00 (-0.16, 0.16) | 0.998 |
| ***Age subgroups*** |  |  |  |  |
| *12-18 years old at endpoint* | *n=2220* |  | *n=1512* |  |
| Parent-child separation < 3m | 0.04 (-0.09, 0.17) | 0.556 | 0.10 (-0.06, 0.25) | 0.224 |
| Parent-child separation ≥ 3m | **-0.11 (-0.22, -0.005)** | **0.041** | -0.07 (-0.20, 0.07) | 0.334 |
| *18-23 years old at endpoint* | *n=636* |  | *n=510* |  |
| Parent-child separation < 3m | 0.07 (-0.13, 0.27) | 0.495 | 0.08 (-0.14, 0.30) | 0.478 |
| Parent-child separation ≥ 3m | 0.07 (-0.14, 0.28) | 0.521 | 0.09 (-0.15, 0.32) | 0.476 |
| ***Residence subgroups*** |  |  |  |  |
| *Rural* | *n=1336* |  | *n=876* |  |
| Parent-child separation < 3m | 0.01 (-0.15, 0.18) | 0.879 | 0.07 (-0.13, 0.26) | 0.503 |
| Parent-child separation ≥ 3m | -0.02 (-0.15, 0.12) | 0.785 | 0.05 (-0.11, 0.22) | 0.513 |
| *Urban/Town* | *n=1520* |  | *n=1146* |  |
| Parent-child separation < 3m | 0.07 (-0.08, 0.23) | 0.341 | 0.12 (-0.06, 0.29) | 0.187 |
| Parent-child separation ≥ 3m | **-0.17 (-0.31, -0.03)** | **0.019** | -0.12 (-0.29, 0.05) | 0.152 |
| ***Maternal education subgroups*** |  |  |  |  |
| *Primary school or below* | *n=1364* |  | *n=871* |  |
| Parent-child separation < 3m | 0.08 (-0.08, 0.25) | 0.325 | 0.18 (-0.02, 0.38) | 0.078 |
| Parent-child separation ≥ 3m | -0.11 (-0.27, 0.05) | 0.165 | -0.04 (-0.23, 0.16) | 0.704 |
| *Middle school or above* | *n=1434* |  | *n=1151* |  |
| Parent-child separation < 3m | 0.03 (-0.12, 0.19) | 0.678 | 0.05 (-0.12, 0.21) | 0.585 |
| Parent-child separation ≥ 3m | -0.04 (-0.17, 0.09) | 0.578 | -0.02 (-0.18, 0.13) | 0.757 |

**^a^** Compared to no parent-child separation group. **^b^** The adjusted models controlled for gender, age in 2010, residence, ethnicity, BMI, paternal and maternal age in 2010, paternal and maternal educational level, family size in 2018 and net household income per capita in 2018, education stage in 2018 and school location. *B* = Unstandardized regression coefficients. CI = Confidence Interval.

**Table S6 Regression analysis of the square of separation duration and depression**

| **Variables** | **Depression scores** | | | | | |
| --- | --- | --- | --- | --- | --- | --- |
|  | *B* | 95% CI | *P* | Adj. *B* ^a^ | 95% CI | *P* |
| Square of separation duration | 0.001 | (-0.0002, 0.002) | 0.101 | 0.002 | (0.0005, 0.003) | **0.005** |
| R^2^ | 0.004 | | | 0.069 | | |
| F | 2.69 | | | 3.11 | | |
| N | 704 | | | 518 | | |

**^a^** Model was adjusted for gender, age in 2010, residence, ethnicity, BMI, paternal and maternal age in 2010, paternal and maternal educational level, family size in 2018 and net household income per capita in 2018. Adj. *B* = Adjusted regression coefficient. CI = Confidence Interval.

**Table S7 Quantile regression of the association between parent-child separation and depression in children during later life after multiple imputation ^a b c^**

| **Variables** | **Different quantile levels of depression** | | | | | | | | | |
| --- | --- | --- | --- | --- | --- | --- | --- | --- | --- | --- |
|  | q25 | | | q50 | | | q75 | | | |
| Parent-child separation | Adj. *B* | 95% CI | *P* | Adj. *B* | 95% CI | *P* | Adj. *B* | 95% CI | *P* |  |
| No separation | Ref. |  |  | Ref. |  |  | Ref. |  |  |  |
| Separation < 3m | -0.24 | (-0.65, 0.17) | 0.257 | -0.46 | (-0.95, 0.04) | 0.069 | -0.003 | (-0.42, 0.41) | 0.988 |  |
| Separation ≥ 3m | 0.40 | (-0.26, 1.07) | 0.233 | 0.46 | (0.09, 0.82) | **0.014** | 0.63 | (0.06, 1.20) | **0.031** |  |
| Pseudo R^2^ | 0.001 | | | 0.016 | | | 0.027 | | |  |
| N | 3829 | | | | | | | | |  |

**^a^** Adjusted models: controlled for gender, age in 2010, residence, ethnicity, BMI, paternal and maternal age in 2010, paternal and maternal educational level, family size in 2018 and net household income per capita in 2018. **^b^** 'q25', 'q50' and 'q75' stand for the quantiles at the 25th, 50th, and 75th percentiles, respectively. **^c^**The CES-D8 scores at q25, q50, and q75 were 2, 4, and 7, respectively. Adj. *B* = Adjusted regression coefficient. CI = Confidence Interval.

**Table S8 Multivariate linear regression of the association of parent-child separation on** **social and academic performance in children during later life after multiple imputation ^a b^**

| **Variables** | **Parent-child relationship** | | | **Peer relationship** | | | **Academic performance** | | | |
| --- | --- | --- | --- | --- | --- | --- | --- | --- | --- | --- |
| Parent-child separation | Adj. *B* | 95% CI | *P* | Adj. *B* | 95% CI | *P* | Adj. *B* | 95% CI | *P* |  |
| No separation | Ref. |  |  | Ref. |  |  | Ref. |  |  |  |
| Separation < 3m | 0.01 | (-0.12, 0.15) | 0.855 | 0.04 | (-0.17, 0.25) | 0.731 | 0.02 | (-0.08, 0.12) | 0.729 |  |
| Separation ≥ 3m | -0.22 | (-0.34, -0.10) | **＜0.001** | -0.19 | (-0.38, -0.01) | **0.044** | -0.07 | (-0.16, 0.02) | 0.112 |  |
| R^2^ | 0.025 | | | 0.003 | | | 0.008 | | | |
| F | 8.20 | | | 1.11 | | | 1.88 | | | |
| N | 3829 | | | 3829 | | | 3829 | | | |

**^a^** Models were adjusted for gender, age in 2010, residence, ethnicity, BMI, paternal and maternal age in 2010, paternal and maternal educational level, family size in 2018. **^b^** For peer relationship, net household income per capita in 2018 was additionally adjusted; for academic performance, net household income per capita in 2018, education stage in 2018, school location and academic stress were additionally adjusted.

**Table S9 Multivariate linear regression of the association of the separation duration on social and academic performance in children during later life after multiple imputation ^a^**

| **Variables** | **Parent-child relationship** | | | **Peer relationship** | | | **Academic performance** | | |
| --- | --- | --- | --- | --- | --- | --- | --- | --- | --- |
|  | Adj. *B* | 95% CI | *P* | Adj. *B* | 95% CI | *P* | Adj. *B* | 95% CI | *P* |
| Separation duration, months | -0.01 | -0.02, -0.003 | **0.014** | -0.02 | -0.03, -0.003 | **0.021** | -0.01 | -0.01, -0.003 | **0.040** |
| R^2^ | 0.058 | | | 0.029 | | | 0.024 | | |
| F | 3.53 | | | 1.69 | | | 1.40 | | |
| N | 704 | | | 704 | | | 704 | | |

**^a^** Models were adjusted for gender, age in 2010, residence, ethnicity, BMI, paternal and maternal age in 2010, paternal and maternal educational level, family size in 2018 and net household income per capita in 2018.

**Table S10 Baseline characteristics in the follow-up and lost to follow-up participants**

| **Baseline characteristics** | **Follow-up**  **(n=3829)** | **Lost to follow-up**  **(n=2881)** | **t/χ2** | ***P*** |
| --- | --- | --- | --- | --- |
| Gender, *n (%)*  Male  Female | 2018 (52.7%)  1811 (47.3%) | 1489 (51.7%)  1392 (48.3%) | 0.685 | 0.408 |
| Age, years, Mean ± SD | 9.2±3.5 | 10.1±3.5 | 10.54 | **＜0.001** |
| Residence, *n (%)*  Rural  Urban/town | 1965 (51.3%)  1864 (48.7%) | 1495 (51.9%)  1386 (48.1%) | 0.216 | 0.642 |
| Ethnicity, *n (%)*  Han  Other | 3384 (88.4%)  445 (11.6%) | 2549 (88.5%)  332 (11.5%) | 0.015 | 0.901 |
| Gestation, months, Mean ± SD | (n=3793)  9.3±0.6 | (n=2816)  9.4±0.6 | 2.509 | 0.012 |
| Birthweight, kg, Mean ± SD | (n=3115)  3.2±0.6 | (n=2262)  3.2±0.6 | 1.206 | 0.228 |
| BMI (kg / m^2^), Mean ± SD | (n=3560)  17.8±4.9 | (n=2678)  17.7±4.7 | 0.690 | 0.490 |
| Paternal age, Mean ± SD | (n=3810)  37.4±5.7 | (n=2849)  38.2±5.9 | 5.623 | **＜0.001** |
| Maternal age, Mean ± SD | (n=3795)  35.5±5.5 | (n=2816)  36.2±5.6 | 4.811 | **＜0.001** |
| Paternal educational level, n (%)  No formal education  Primary school  Middle school  High school/Technical school  College or higher | (n=3762)  578 (15.4%)  973 (25.9%)  1566 (41.6%)  463 (12.3%)  182 (4.8%) | (n=2804)  426 (15.2%)  709 (25.3%)  1082 (38.6%)  347 (12.4%)  240 (8.6%) | 38.543 | **＜0.001** |
| Maternal educational level, *n (%)*  No formal education  Primary school  Middle school  High school/Technical school  College or higher | (n=3753)  978 (26.1%)  1034 (27.6%)  1299 (34.6%)  294 (7.8%)  148 (3.9%) | (n=2781)  774 (27.8%)  661 (23.8%)  896 (32.2%)  250 (9.0%)  200 (7.2%) | 47.613 | **＜0.001** |
| Parental marital status, *n (%)*  In marriage  Divorced/Single/Widowed | (n=3779)  3687 (97.6%)  92 (2.4%) | (n=2807)  2711 (96.6%)  96 (3.4%) | 5.641 | **0.018** |
| Parent-child separation, *n (%)*  No separation  Separation < 3 months  Separation ≥ 3months | 3125 (81.6%)  306 (8.0%)  398 (10.4%) | 2263 (78.5%)  216 (7.5%)  402 (14.0%) | 19.907 | **＜0.001** |
